# Addressing the Gaps in the Vitamin B12 Deficiency 2024 NICE Guidelines: Highlighting the Need for Better Recognition, Diagnosis, and Management of Pernicious Anaemia

**DOI:** 10.1038/s41430-025-01583-4

**Published:** 2025-02-21

**Authors:** Alfie Thain, Kathryn Hart, Kourosh R. Ahmadi

**Affiliations:** School of Biosciences, Faculty of Health & Medical Sciences University of Surrey, Guildford, UK

**Keywords:** Health policy, Autoimmune diseases, Gastritis

## Abstract

The 2024 NICE guidelines on vitamin B_12_ deficiency have significant implications for the diagnosis and management of pernicious anaemia (PA), the commonest non-dietary cause of such deficiency. This perspective discusses the guidelines *in relation to PA itself*, suggests that clearer diagnostic protocols are required, and calls for clinician education to improve the patient journey for those with PA.

## Introduction

Pernicious anaemia (PA) is an underdiagnosed complex autoimmune condition characterised by vitamin B_12_ (B_12_) malabsorption. This occurs secondary to gastric atrophy which leads to a reduction or absent production of intrinsic factor (IF) [1]. While PA is linked to the end stages of autoimmune gastritis (AIG), they are not synonymous [2]. PA remains largely under-researched in clinical practice, contributing to gaps in diagnosis, management, and patient care. Its management typically involves regular and lifelong intramuscular injections of hydroxocobalamin. An injection frequency of 1 mg every 2–3 months is often ‘recommended’. However, this dosing schedule does not have a strong evidence base, and many individuals with PA / B_12_ deficiency / PA report requiring more frequent injections for adequate symptom relief [3]. Furthermore, recent data, which is consistent with prior work by Hooper et al. [4], from a large survey we conducted on over 1100 PA patients shows that 40% of PA patients wait two years or longer for diagnosis, further complicating patient outcomes and satisfaction (Thain et al., 2024, DOI: 10.1101/2024.08.30.24312837).

The 2024 National Institute for Health and Care Excellence (NICE) guidelines on B_12_ deficiency raise important questions about PA, particularly regarding its nomenclature, diagnostic criteria, and appropriate follow-up care [5]. Here we review the NICE guidelines, with a specific focus on their implications for PA, and highlights areas for improvement in addressing the needs of such individuals.

## Renaming of pernicious anaemia as autoimmune gastritis

While the guideline has several strengths, the proposal to replace the term ‘pernicious anaemia’ with ‘autoimmune gastritis’ is, in our opinion, a notable limitation. PA is considered to develop in advanced stages of autoimmune gastritis (AIG) rather than being entirely synonymous with it [6]. It is important to acknowledge that PA and AIG represent different stages of the same disease continuum. Autoimmune gastritis, characterised by advanced atrophy of the gastric oxyntic mucosa with a spared antrum, forms the pathological basis for PA. However, PA specifically refers to the clinical manifestation associated with IF deficiency and subsequent B_12_ malabsorption. Therefore, these conditions should not be referred to as identical entities but recognised as distinct stages within the natural history of the same disease. This change could exacerbate current confusion and add further uncertainty regarding the diagnosis and management of PA. The guidelines state, “*However, pernicious anaemia in its true sense (that is, life-threatening anaemia) is now extremely rare because of developments in testing and treatment for, and greater awareness of, vitamin B*_*12*_ *deficiency*.”

In one sense, this statement is correct: the term ‘pernicious anaemia,’ when taken literally, implies a severe, life-threatening form of anaemia, which is uncommon in modern practice due to improvements in testing and treatment, such severe manifestations are typically prevented, as the condition is more likely to be diagnosed earlier or treated with B_12_. However, changing the name overlooks the broader clinical context of PA, which is not just an issue of anaemia but rather a condition characterised by the autoimmune-mediated destruction of parietal cells leading to IF deficiency and lifelong B_12_ malabsorption [1]. Three other points warrant mentioning. First, there is also a separate autoimmune-mediated attack on IF that will lead to PA. Second, not all AIG diagnoses translate to PA. PA occurs in only about 15–20% of AIG patients, highlighting the inaccuracy of using AIG as a substitute for PA [7, 8]. The life-course of PA development has not been studied, but there is sufficient information to highlight that the timelines are highly varied among patients. For instance, some individuals may develop PA as early as 1 year after the onset of AIG, while others may not progress to PA until 15 or more years after AIG onset [9]. Thus, whilst PA is acknowledged as a consequence of AIG, conflating the two terms risks oversimplifying the disease process and ignoring the clinical specificity of PA as an advanced manifestation. Third, over 50% of individuals with symptomatic B deficiency test negative for IF or parietal cell auto-antibodies, and the true prevalence of AIG in the UK is not known, as individuals with B deficiency rarely undergo endoscopy.

Whilst renaming PA as AIG can add confusion and uncertainty, the name PA has its own shortcomings, which is perhaps why NICE proposed to change it. The term ‘pernicious anaemia’ suggests that anaemia is a defining characteristic of the disease. However, anaemia is present in only 15–20% of PA diagnoses [2, 10]. Practitioners unfamiliar with PA are commonly misled by the term ‘anaemia’, as all ICD-10 (D51.0-9) codes for B deficiency incorrectly include the term anaemia. The term ‘pernicious’ is also an anachronism, predating the 1948 discovery of B as an effective treatment. It was first coined by Anton Biermer in 1871 to describe the fatal anaemia cases seen prior to the discovery of effective treatments. It is important to note that even though anaemia occurs in a minority of PA patients, this represents an important subgroup of patients that requires a different management approach. The current guidelines do not address how to specifically diagnose and manage this subgroup of patients, representing a significant gap in knowledge and warranting further research.

The definition and our understanding of the condition has evolved significantly since Biermer’s original description. We now know that PA often presents with predominantly neurological symptoms [3, 11]. Therefore, if a name change is warranted, it should be one that better reflects the current clinical presentation of the disease.

The persistent use of the term ‘pernicious anaemia’ in both clinical practice (primary, secondary and tertiary care) and *state-of-the-art* research highlights a disconnect between these guidelines and current clinical practice. It is important to highlight that the adoption of new and more appropriate terminology would require universal acceptance by clinicians, carers and patients who would have lived with the name PA for most of their lives. While changing established medical terminology may take decades—potentially one to two generations of doctors—before it is fully adopted in clinical language, emphasising that such a change, if it were to happen, would be gradual and require effective dissemination.

However, a name change could offer significant advantages by reflecting the current understanding of PA and encouraging clinicians to investigate neurological symptoms associated with PA [11]. This would be achieved through a concerted effort to educate doctors by incorporating this knowledge into the curriculum, which will dispel misconceptions commonly ascribed to PA patients by doctors and promote a deeper understanding of PA’s complexity.

## Diagnosing PA: the commonest non-nutritional cause of vitamin B deficiency

Given how NICE guidelines are used in practice, we find that the guidelines lack specific advice on the clinical scenarios in which practitioners should suspect AIG and PA. While they recommend considering an anti-intrinsic factor autoantibody test if AIG is suspected, the guidelines do not adequately explain why general practitioners should suspect an autoimmune basis beyond typical gastrointestinal symptoms. The presentation of AIG is highly variable, and looking only for gastrointestinal symptoms is insufficient as these present only in the later stages or not at all in most patients [6, 12]. Intrinsic factor auto-antibodies (IF-Ab) perform poorly as a stand-alone diagnostic marker for PA. Although they are highly specific, they have low sensitivity and present in less than 50% of PA patients [13]. The guidelines acknowledge that a negative IF-Ab test does not rule out AIG or PA, and they suggest additional investigations when clinical suspicion remains. They further state that these should include anti-gastric parietal cell antibody (PC-Ab) tests, gastrin level measurements, CobaSorb tests, or gastroscopy with gastric body biopsy. However, despite their high sensitivity, PC-Ab has poor specificity and is present in other autoimmune conditions (e.g. autoimmune thyroid diseases [14]) and, more generally, in approximately 10% of the general population [15]. However, the uncertainty with this is whether it presents a pre-vitamin B12 deficiency or PA, where individuals are not yet symptomatic or are symptomatic but have a serum B12 value within the normal reference ranges. Therefore, a positiveresult will not definitively indicate PA or AIG. Additionally, PC-Ab might test positive in cases of Helicobacter pylori (H. pylori)-related chronic superficial gastritis, further complicating its diagnostic utility [16].

Furthermore, gastrin levels are influenced by multiple factors, including use of proton pump inhibitors (PPIs), which are commonly prescribed among older adults [17]. The use of PPIs can increase plasma gastrin levels by 3–5 times, dependent on treatment duration, necessitating suspension before testing, yet the 2024 guidelines do not address this [18]. Additionally, gastrin levels may also be elevated by *H. pylori* infection. Finally, commercially available kits often measure only one gastrin form, potentially yielding false negatives. Dottori et al. propose a diagnostic pathway for patients suspected of having AIG, which we would recommend to guide the diagnosis of both PA and AIG [19].

Once AIG and/or PA are suspected and/or test results are positive, individuals should undergo a gastroscopy/endoscopy with histological sampling to determine the presence of AIG [20]. This is essential and routinely practised in many countries around the world for confirmation of PA diagnosis and for the atrophy to be graded for risk stratification in the case of the development of gastric lesions [21]. However, this procedure is rarely performed for PA patients in the UK. Although a positive result for IF-ABs can confirm a diagnosis of PA, gastroscopy remains important for confirming AIG and assessing the degree of gastric atrophy. Results from a recent study found that only 9% of PA patients were offered an gastroscopy/endoscopy at diagnosis, highlighting an important gap in the diagnostic approach for PA (Thain et al., 2024, DOI: 10.1101/2024.08.30.24312837). A previous survey also reported that 131 out of 561 (23.3%) patients had undergone endoscopy following a diagnosis of PA, with biopsies taken in 108 of the endoscopies [22]. While the exact reasons are not well-documented, it is likely due to a combination of factors, including cost/limited availability of resources, and poor knowledge of the disease. Additionally, some patients may be hesitant to undergo the procedure due to concerns about its invasiveness or perceived risks despite it being a procedure with minimal risk [23].

## There are also several positives for PA patients from the 2024 NICE guidelines

The guidelines’ emphasis on a symptom-based approach to treatment is an urgent and necessary shift that could positively influence current practice. They also stress the importance of individualised care, suggesting that B_12_ replacement dosage, frequency, and delivery method may need to be tailored to ensure treatment efficacy. However, many patients require treatment more frequently than current guidelines suggest. Survey data indicates that up to 50% of patients require more frequent injections to manage their symptoms effectively (Thain et al., 2024, DOI: 10.1101/2024.08.30.24312837). This is consistent with findings from Hooper et al., where 65% of patients received B_12_ injections according to current guidelines (every 2–3 months) [4]. Further qualitative evidence from interviews also highlights that many PA patients considered the current guidelines too restrictive, with symptoms reoccurring well before the next planned injection [24].

The guidelines highlight key research recommendations aligning with the recent *James Lind Alliance priority-setting partnership* priorities [25]. One of these priorities is improving our understanding of the clinical, quality-of-life and cost-effectiveness of self-care for PA patients - including self-administering B_12_ injections - which could empower patients to manage their symptoms and reduce NHS costs. While the clinical utility and patient perception of self-administration have not been widely studied, results from Thain et al. and Tyler et al. indicate that more than a third of patients with PA or B12 deficiency self-inject, varying degrees of authorisation from healthcare professionals. Key drivers included dissatisfaction with treatment frequency and perceived lack of clinician knowledge, with 80% citing improved quality of life as their primary motivation for self-injecting [26].

Another critical research question highlighted by the NICE guidelines is determining what should be included in follow-up reviews for individuals with B_12_ deficiency, including those with AIG and PA. Follow-up care is negligible for UK PA/AIG patients, with no regular reviews of treatment efficacy, symptom/disease progression, and concomitant disease risk assessment. Existing data shows there is a significantly (2–8 times) higher risk of gastric cancer among individuals with AIG, chronic atrophic gastritis, and PA [27]. Additionally, there is an increased risk of gastric type 1 neuroendocrine tumours, as highlighted in recent work by Rugge et al. [28]. While some countries have routine monitoring and screening guidelines, such as the EU’s Management of Epithelial Precancerous Conditions and Lesions in the Stomach (MAPS), the UK lacks such a system [29]. This gap in monitoring was also identified as an area for action by the James Lind Alliance in 2020, supporting the need for regular testing due to the increased risk of gastric cancer.

## Conclusions

The publication of the 2024 NICE guidelines on B_12_ deficiency represents progress but falls short by proposing to rename ‘pernicious anaemia’ with ‘autoimmune gastritis.’ This change does not adequately address the distinct nature of PA as an advanced stage of AIG, characterised by IF deficiency requiring lifelong B_12_ treatment. Although severe and life-threatening anaemia due to PA is rare today, PA remains a specific condition that requires recognition and appropriate management. The guidelines currently lack detailed protocols for diagnosing PA, especially in patients without typical gastrointestinal symptoms, and rely on tests with limited sensitivity and specificity. Despite these limitations, the guidelines do highlight the importance of a symptom-based approach and prioritise critical research initiatives to address many of the current shortcomings associated with the patient journey. Future revisions should better distinguish PA from AIG, incorporate comprehensive diagnostic pathways that can be implemented in practice, and emphasise educating medical students and professionals to improve diagnosis, effective management and prognosis of PA.

## References

[1] Doniach D, Roitt IM, Taylor KB. Autoimmune phenomena in pernicious anaemia. BMJ. 1963;1:1374–9. 14028630 10.1136/bmj.1.5342.1374 PMC2124044

[2] Rustgi SD, Bijlani P, Shah SC. Autoimmune gastritis, with or without pernicious anemia: epidemiology, risk factors, and clinical management. Ther Adv Gastroenterol. 2021;14:175628482110387. 10.1177/17562848211038771 PMC8414617 34484423

[3] Wolffenbuttel BH, Owen PJ, Ward M, Green R. Vitamin B 12. BMJ. 2023;383:e071725. 37984968 10.1136/bmj-2022-071725 PMC10658777

[4] Hooper M, Hudson P, Porter F, McCaddon A. Patient journeys: diagnosis and treatment of pernicious anaemia. Br J Nurs. 2014;23:376–81. 24732991 10.12968/bjon.2014.23.7.376

[5] Vitamin B12 deficiency in over 16s: diagnosis and management. NICE guideline NG239. 2024. 38713783

[6] Lenti MV, Rugge M, Lahner E, Miceli E, Toh BH, Genta RM, et al. Autoimmune gastritis. Nat Rev Dis Prim. 2020;6:56. 32647173 10.1038/s41572-020-0187-8

[7] Marignani M, Fave DG, Mecarocci S, Bordi C, Angeletti S, D’Ambra G, et al. High prevalence of atrophic body gastritis in patients with unexplained microcytic and macrocytic anemia. Am J Gastroenterol. 1999;94:766–72. 10086664 10.1111/j.1572-0241.1999.00949.x

[8] Lahner E, Annibale B. Pernicious anemia: New insights from a gastroenterological point of view. World J Gastroenterol. 2009;15:5121. 19891010 10.3748/wjg.15.5121 PMC2773890

[9] Irvine WJ, Cullen DR, Mawhinney H. Natural history of autoimmune achlorhydric atrophic gastritis A 1-15-year follow-up study. Lancet. 1974;304:482–5. 10.1016/s0140-6736(74)92013-3 4137076

[10] Song IC, Lee HJ, Kim HJ, Bae SB, Lee KT, Yang YJ, et al. A multicenter retrospective analysis of the clinical features of pernicious anemia in a Korean population. J Korean Med Sci. 2013;28:200. 23400269 10.3346/jkms.2013.28.2.200 PMC3565130

[11] Healton EB, Savage DG, Brust JCM, Garrett TJ, Lindenbaum J. Neurologic aspects of cobalamin deficiency. Medicine. 1991;70:229–45. 1648656 10.1097/00005792-199107000-00001

[12] Orgler E, Dabsch S, Malfertheiner P, Schulz C. Autoimmune gastritis: update and new perspectives in therapeutic management. Curr Treat Options Gastroenterol. 2023;21:64–77.

[13] Marzinotto I, Dottori L, Baldaro F, Dilaghi E, Brigatti C, Bazzigaluppi E, et al. Intrinsic factor autoantibodies by luminescent immuno-precipitation system in patients with corpus atrophic gastritis. J Transl Autoimmun. 2021;4:100131. 35005595 10.1016/j.jtauto.2021.100131 PMC8716657

[14] Tozzoli R, Kodermaz G, Perosa AR, Tampoia M, Zucano A, Antico A, et al. Autoantibodies to parietal cells as predictors of atrophic body gastritis: A five-year prospective study in patients with autoimmune thyroid diseases. Autoimmun Rev. 2010;10:80–3. 20696284 10.1016/j.autrev.2010.08.006

[15] Lahner E, Norman GL, Severi C, Encabo S, Shums Z, Vannella L, et al. Reassessment of intrinsic factor and parietal cell autoantibodies in atrophic gastritis with respect to cobalamin deficiency. Am J Gastroenterol. 2009;104:2071–9. 19491828 10.1038/ajg.2009.231

[16] Malfertheiner P, Camargo MC, El-Omar E, Liou JM, Peek R, Schulz C, et al. Helicobacter pylori infection. Nat Rev Dis Prim. 2023;9:19. 37081005 10.1038/s41572-023-00431-8 PMC11558793

[17] Waldum HL, Kleveland PM, Fossmark R. Upper gastrointestinal physiology and diseases. Scand J Gastroenterol. 2015;50:649–56. 25857514 10.3109/00365521.2015.1009157

[18] Rehfeld JF. Gastrin and the moderate hypergastrinemias. Int J Mol Sci. 2021;22:6977. 34209478 10.3390/ijms22136977 PMC8269006

[19] Dottori L, Pivetta G, Annibale B, Lahner E. Update on serum biomarkers in autoimmune atrophic gastritis. Clin Chem. 2023;69:1114–31. 37680186 10.1093/clinchem/hvad082

[20] Sipponen P, Price AB. The Sydney System for classification of gastritis 20 years ago. J Gastroenterol Hepatol. 2011;26:31–4. 21199511 10.1111/j.1440-1746.2010.06536.x

[21] Esposito G, Dottori L, Pivetta G, Ligato I, Dilaghi E, Lahner E. Pernicious anemia: the hematological presentation of a multifaceted disorder caused by cobalamin deficiency. Nutrients. 2022;14:1672. 35458234 10.3390/nu14081672 PMC9030741

[22] Pritchard DM, Hooper M. Letter: gastric cancer and pernicious anaemia – only a minority of UK pernicious anaemia patients have had a gastroscopy. Aliment Pharm Ther. 2016;43:1106–7. 10.1111/apt.13595 27072321

[23] Waddingham W, Kamran U, Kumar B, Trudgill NJ, Tsiamoulos ZP, Banks M. Complications of diagnostic upper Gastrointestinal endoscopy: common and rare – recognition, assessment and management. BMJ Open Gastroenterol. 2022;9:e000688. 36572454 10.1136/bmjgast-2021-000688 PMC9806027

[24] Seage CH, Glover E, Mercer J. Receiving a diagnosis of pernicious anemia: exploring experiences of relationships with health professionals. J Patient Exp. 2020;7:766–70. 33294613 10.1177/2374373519883497 PMC7705842

[25] Staley K, Ahmadi KR, Carter K, Cowan K, Seage H, Visser P, et al. Research priorities in pernicious anaemia: James Lind Alliance Priority Setting Partnership. BMJ Open. 2022;12:e065166. 36002205 10.1136/bmjopen-2022-065166 PMC9413171

[26] Tyler N, Hodkinson A, Ahlam N, Giles S, Zhou A, Panagioti M. Patient safety, self-injection, and B12 deficiency: a UK cross-sectional survey. Br J Gen Pract. 2022;72:e891–8. 36192360 10.3399/BJGP.2021.0711 PMC9550316

[27] Vannella L, Lahner E, Osborn J, Annibale B. Systematic review: gastric cancer incidence in pernicious anaemia. Aliment Pharm Ther. 2013;37:375–82. 10.1111/apt.12177 23216458

[28] Rugge M, Genta RM, Malfertheiner P, Dinis-Ribeiro M, El-Serag H, Graham DY, et al. RE.GA.IN.: the Real-world Gastritis Initiative–updating the updates. Gut. 2024 Feb 21;gutjnl-2023-331164. 10.1136/gutjnl-2023-331164 38383142

[29] Pimentel-Nunes P, Libânio D, Marcos-Pinto R, Areia M, Leja M, Esposito G, et al. Management of epithelial precancerous conditions and lesions in the stomach (MAPS II): European Society of Gastrointestinal Endoscopy (ESGE), European Helicobacter and Microbiota Study Group (EHMSG), European Society of Pathology (ESP), and Sociedade Portuguesa de Endoscopia Digestiva (SPED) guideline update 2019. Endoscopy. 2019;51:365–88. 30841008 10.1055/a-0859-1883

